# Insight and its relationship with stigma in psychiatric patients

**DOI:** 10.4103/0972-6748.57858

**Published:** 2009

**Authors:** Deepak K. Mishra, Sarika Alreja, K. S. Sengar, Amool R. Singh

**Affiliations:** Department of Clinical Psychology, Ranchi Institute of Neuro Psychiatry and Allied Sciences (RINPAS), Kanke, Ranchi - 834 006, India

**Keywords:** Insight, Psychiatric patients, Stigma

## Abstract

**Background:**

The literature on insight has paid insufficient attention to the social experiences that are associated with receiving and endorsing a diagnosis of mental illness. The psychological and behavioral commitments associated with insight extend beyond agreeing with a diagnosis and accepting treatment to include taking on the identity of an individual diagnosed with mental illness. This study sought to examine the relationship between insight and stigma in psychiatric patients.

**Materials and Methods:**

Cross-sectional assessment of insight and stigma was done using the system adopted by Kaplan and Sadock in their comprehensive textbook of psychiatry and Felt Stigma Scale in 100 psychiatric patients (40 patients suffering from Bipolar affective disorder, 30 Schizophrenics, 20 Substance dependents and 10 with Obsessive Compulsive disorder).

**Results:**

It was found that the level of stigma felt by patients with insight was significantly higher than that felt by patients without insight.

**Conclusion:**

Though there is a certain extent of stigma present in patients without insight, as is expected, the level of stigma increases as the patients develop insight.

In psychiatry, the term *insight* is used to refer to the capacity to recognize that one has an illness that requires treatment (Ghaemi, 1997). Research suggests that individuals diagnosed with psychotic illnesses are more likely than any other patient group to be assessed as having poor insight (Amador *et al*., 1994; Lysaker *et al*., 2002). Mental health professionals view insight as integral to achieving treatment compliance and promoting positive social and health outcomes for diagnosed individuals (McEvoy, 1998; McGorry & McConville, 1999). Yet research shows that interventions to promote insight have not led to improved receptivity to treatment or adherence to treatment programs (Beck-Sander, 1998; O’Donnell *et al*., 2003). In fact, recent work suggests that insight may be a clinical phenomenon that is independent of beliefs about the usefulness of medical treatment (Linden & Godemann, 2007). In addition, the search for positive outcomes from insight has revealed negative outcomes, particularly in the areas of quality of life and self-esteem (Kravetz *et al*., 2000; O’Mahony, 1982; Schwartz, 1998; Warner *et al*., 1989). The concept of insight is problematic because it merges several aspects of the mental illness experience that may not belong together. An examination of the theoretical and empirical literatures in the area reveals a m élange of ideas about awareness of illness, acceptance of illness, willingness to take medication or other treatment, and endorsement of other expectations that are applied to people with mental disorders (Kravetz *et al*., 2000). There is one consistency; judgments of insight are always based on the extent to which patients endorse a biomedical explanation for illness. Although insight usually describes adherence to a particular belief system about mental illness, some assume that lack of insight reflects the absence of complex, reflective thought (White *et al*., 2000). Poor insight may be attributed to neuropsychological deficit, unremitting psychopathology, or unsophisticated ego defense mechanisms (Ghaemi, 1997). In contrast, good insight is presumed to be the outcome of an appropriate developmental or restorative process that transforms a previously unaware or highly defensive patient into one who is aware and compliant. The high insight/high functioning and low insight/dysfunctional distinctions seem clear in theory, but reality is not as easily categorized. The patients that we see with high insight are not always functioning well, and the patients that we see with low insight are not always functioning poorly. Both research literature and clinical experience suggest that a patient’s acceptance of the medical explanation for the experiences of mental illness does not tell us everything we need to know about how they are coping with diagnosis.

Stigmatization involves a separation of individuals labeled as different from “us” who are believed to possess negative traits, resulting in negative emotional reactions, discrimination and status loss for the stigmatized person (Link *et al*., 2004). Stigmatization of individuals diagnosed as having serious mental illness has been observed across the world and the family members who help care for them also feel stigmatized as a result of their association with the loved one with mental illness (Phelan *et al*., 1998; Phillips *et al*., 2002; Struening *et al*., 2001; Thara and Srinivasan; 2000). Studies on psychiatric stigma have often focused on public attitudes. Because these collective attitudes vary in their impact on individuals, and stigma is ultimately an inner subjective experience, they provide at best an approximate guide to how stigma causes difficulties to individuals with mental illness. In contrast, understanding patients’ subjective experiences of stigma attenuates us to what is at stake in their local lived world, i.e. the everyday non-trivial interpersonal transactions involving family members, partners, friends and colleagues (Kieinman and Kleinman, 1977).

The insight literature has paid insufficient attention to the social experiences that are associated with receiving and endorsing a diagnosis of mental illness. Insight involves taking on a new identity that changes the way individuals see themselves, and the ways that others see them. The insight concept is changed by recognition of its ties to identity processes that extend beyond the biomedical explanation for illness. Insight certainly reflects the extent to which individuals agree with their doctors, but it is also an indication of the extent to which individuals are willing to identify themselves as part of a group of people that are similarly affected. Consequently, the psychological and behavioral commitments associated with insight extend beyond agreeing with a diagnosis and accepting treatment to include taking on the identity of an individual diagnosed with mental illness. The expectations that individuals have for post-diagnosis identity may be extremely constricted or highly elaborated, based on the spectrum of patient identity representations that are known to them. The expectations they have for group identification with the community of mentally ill people are likely to be influenced by previous knowledge of mental illness and interactions they have had with family and friends, healthcare professionals, other patients, and society as a whole. Therefore, the identity shifts precipitated by diagnosis are affected by information and experiences embedded in the social context.

This conceptualization suggests that an intersection of individual and social processes encourages or discourages expressing beliefs that correspond to good insight. These ideas clearly require empirical validation, and recent work by Lysaker *et al*. (2006) is intriguing in this regard. They interviewed 75 patients with schizophrenia spectrum disorders to explore how self-stigma might explain the paradoxical links between greater insight, better functional outcomes, and poorer subjective wellbeing. Their cluster analysis of data from measures assessing insight and internalized stigma identified three groups: low insight/mild stigma, high insight/minimal stigma, and high insight/moderate stigma. Their attempt to compare the groups on measures of quality of life, self-esteem, and hope revealed that the high insight/low stigma group had significantly better interpersonal functioning. In contrast, increases in vulnerability to self-stigma demonstrated in the high insight/moderate stigma group corresponded to reports of less self-esteem and less hope. The analyses in the study did not reveal anything further about individuals demonstrating other configurations of insight and self-stigma.

## MATERIALS AND METHODS

### Sample

The present study was a cross-sectional study for which a sample consisting of 100 patients from the inpatient and outpatient services of the Ranchi Institute of Neuro Psychiatry and Allied Sciences was taken using purposive sampling. Of the 100 patients 30 were suffering from Schizophrenia, 30 from Bipolar Affective Disorder Manic type, 10 from Bipolar Affective Disorder Depressive type, 20 were Substance Dependents and 10 were Obsessive Compulsive Disorder patients. Subjects were between the age range of 25-35 years and were educated minimum up to primary level. Both male and female subjects were taken for the study. Patients with any other neurological disorder/major physical illness were excluded. All subjects were cooperative and gave verbal consent for the study. Sample characteristics are given in Table 1.

**Table 1:** Socio-demographic and clinical characteristics of the sample

Variables	Insight present N = 50 M + SD/N (%)	Insight absent N = 50 M + SD/N (%)	t/x	*P*
Age	32.36 + 7.72	29.0 + 6.43	2.363	0.020*
Sex			0.00	1.00
Male	44	44		
Female	6	6		
Education			4.77	0.19
Primary	3	10		
Matric	19	19		
Intermediate	15	11		
Graduate	13	10		
Marital status			0.372	0.542
Single	22	19		
Married	28	31		
Occupation			5.523	0.238
Service	5	3		
Business	23	14		
Student	1	2		
Others	6	6		
Unemployed	15	25		
Religion			5.186	0.075
Hindu	45	41		
Muslim	3	9		
Christian	2	0		
Socioeconomic status			5.033	0.081
Lower	8	13		
Middle	42	34		
Upper	0	3		
Domicile			0.978	0.613
Rural	16	20		
Semi-urban	17	13		
Urban	17	17		
Diagnosis			20.00	0.00**
Schizophrenia	15	15		
BAD M	15	15		
BAD D	0	10		
Substance dependence	10	10		
OCD	10	0			

* Significant at 0.5 level;

** Significant at 0.1 level.

### Tools

#### Socio-demographic and clinical data sheet

Socio-demographic and clinical details were collected on a socio-demographic and clinical data sheet especially designed for the study. It includes various socio-demographic variables (i.e. age, sex, marital status, family type, residence, education and religion etc.) clinical variables (i.e. diagnosis, total duration of illness).

#### Brief psychiatric rating scale (Overall et al., 1963)

This scale was administered to assess the severity of psychiatric symptoms.

#### Felt stigma scale (King et al., 2007)

Developed with the help of the Self-Esteem Scale published in the *British Journal of Psychiatry*, March 2007. It constitutes of a total of 28 items. Fifteen appropriate items to the socio-cultural aspects of the sample, were selected for the study. The scoring was done on a three-point scale: strongly agree, agree and disagree. Eleven items were positively worded and four were negatively worded so the scores reversed for negatively worded items. The minimum score that can be obtained is 15 and maximum is 45. The higher the score, the higher is the felt stigma. For computing levels of felt stigma the scores were also measured in three levels, 15-25 as low, 26-35 as medium and 36-45 as high felt stigma.

#### Insight

The system adopted by Kaplan and Sadock in their *Comprehensive Textbook of Psychiatry* (2000) was used to grade the patient’s insight level.

### Procedure

Socio-demographic and clinical information was collected using the Socio-demographic and Clinical Data Sheet. Information was gathered from reliable sources. Participants were selected from the inpatient and outpatient services of the Ranchi Institute of Neuro Psychiatry and Allied Sciences. Participants who fulfilled the inclusion/exclusion criteria were screened for severity using the Brief Psychiatric Rating Scale. Subjects found in the range of mild to moderate level of severity on this scale participated in the study. Kaplan and Sadock’s (2000) system was used to grade patient’s insight level. Insight was considered to be absent when it was found to be below Grade III. Patients found to be having insight above Grade III level were considered as having insight. To assess the perceived stigma Felt Stigma Scale (King *et al*., 2007) was administered.

### Statistical analysis

The Statistical Package for Social Sciences (SPSS) Version 10.0 was used for statistical analysis. Data of the present study is described using t-test for continuous variables and Chi-square test for categorical variables.

## RESULTS AND DISCUSSION

The study aimed to compare the felt stigma and its relationship with insight among patients attending inpatient and outpatient services of RINPAS. For this purpose we tried to match both the groups (i.e. with insight and without insight) in various socio-demographic and clinical variables. Both the groups were matched for sex, education, marital status, occupation, religion, domicile, and socioeconomic status but differed significantly for age. In the clinical variables, both the groups differed significantly for the diagnostic group. Our study revealed that both the groups (i.e. with insight and without insight) have significantly different levels of felt stigma [Table 2].

**Table 2:** Stigma score and presence of insight

Variable	N	Stigma score M + SD	T	*P*
Insight			30.297	0.00**
Present	50	39.90 ± 4.81		
Absent	50	16.52 ± 2.56		

** Significant at. 01 level

Individuals diagnosed with mental illness can only occupy post-diagnosis identities that are known and available to them. Knowledge of a range of post-diagnosis identities depends on exposure to heterogeneity of experience, perhaps through dialogues with mental health professionals, contacts with other diagnosed individuals, and more diverse portrayals of mentally ill people in education and the media. Even with that knowledge, however, mobility across post-diagnosis identities, and other social identities, is not equally available. Some individuals may function in social environments that constrain choices for group identification, forcing them into situations of social isolation or binding them to groups that may or may not meet their needs. Therefore, awareness of identity options does not guarantee access to them. Furthermore, variations in individual characteristics like personality, creativity, self-confidence and life opportunities may alter the capacity to enact various post-diagnosis identities, or juxtapose them with other social identities. Similarly, periods of illness and recovery that may produce fluctuations in cognitive function, social skills, and expression of paranoia, depression, and other symptoms can alter capacity for group identifications and withstanding stigma. Access to specific post-diagnosis identities is likely determined by individual, social, and illness-related factors that can change, contributing to unfixed relationships between insight, treatment compliance, and psychosocial outcomes. This conceptualization encourages the replacement of dichotomies of good insight versus poor insight with consideration of internal and external resources that might promote the enactment of various post-diagnosis identities. If movement among post-diagnosis identities is expected and perhaps desirable, then it becomes important to ensure that individuals are equipped with the internal and external resources to shift identities as circumstances demand.

## CONCLUSION

Findings indicate that though there is certain amount of stigma present in patients without insight, as is expected, the level of stigma increases as the patients develop insight into their illness. Future empirical work may further clarify the connections between awareness of illness and both individual and social processes that influence psychosocial outcomes for people diagnosed with mental illnesses. At the same time, the study reveals how much more is still unknown. Identifying cross-sectional connections between insight, self-stigma, hope, self-esteem, and social functioning cannot tell us all that we want to know about the longitudinal process of living with diagnosis. Our patients grapple with constructing a personal narrative that includes the experiences of mental illness, integrating a post-diagnosis identity with other social identities, and negotiating these identities in a social context that may not accommodate one or more of them.
